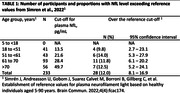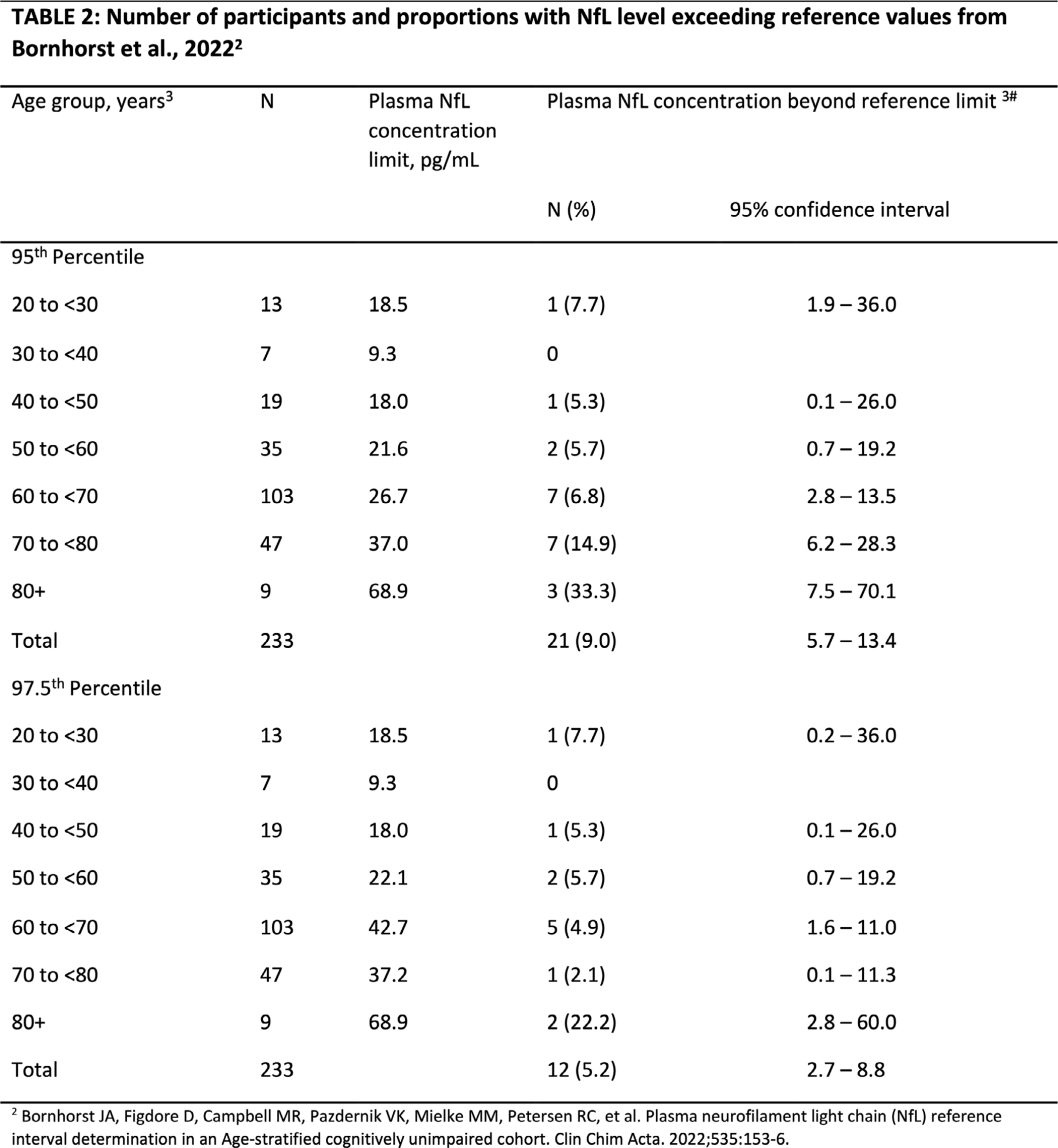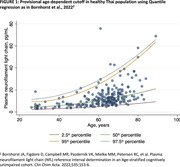# The generalizability of proposed reference values of plasma neurofilament light chain level in Thai healthy population

**DOI:** 10.1002/alz.092169

**Published:** 2025-01-09

**Authors:** Paramee Supaksirakol, Pasin Hemachudha, Akarin Hiransuthikul, Watayuth Luechaipanit, Thanaporn Haethaisong, Adipa Chongsuksantikul, Thiravat Hemachudha, Poosanu Thanapornsangsuth

**Affiliations:** ^1^ Faculty of Medicine, Chulalongkorn University, Bangkok, Bangkok Thailand; ^2^ Elderly Health Care Center, Queen Savang Vadhana Memorial Hospital, Sriracha, Chonburi Thailand; ^3^ Thai Red Cross Emerging Infectious Diseases Health Science Centre, King Chulalongkorn Memorial Hospital, Bangkok Thailand; ^4^ Chula Neuroscience Centre, Bangkok, Bangkok Thailand; ^5^ Chula Neuroscience Center, King Chulalongkorn Memorial Hospital, Bangkok Thailand; ^6^ King Chulalongkorn Memorial Hospital, Bangkok Thailand; ^7^ King Chulalongkorn Memorial Hospital The Thai Red Cross Society, Bangkok Thailand; ^8^ Faculty of Medicine, Chulalongkorn University, Bangkok Thailand

## Abstract

**Background:**

Aging is an established confounding factor influencing the plasma levels of neurofilament light chain (NfL). While age‐specific cutoff values for NfL in healthy Caucasian populations have been documented, the potential variations in ethnically and socioeconomically underrepresented populations remain underexplored. This study aims to evaluate the acceptability of proposed NfL cutoff values in the healthy Thai population.

**Method:**

The study included 233 healthy participants aged 18 years and above, drawn from the Comprehensive Geriatric Clinic at King Chulalongkorn Memorial Hospital, the Cognitive Aging Cohort, and controlled participants from various studies in Bangkok, Thailand. Plasma NfL levels were quantified using the single molecule array (Simoa®) NF‐light™ Advance kit. Utilizing reference threshold from previous studies (Simrén et al., 2022 and Bornhorst et al., 2022), the proportion of participants with NfL levels exceeding the proposed cutoff values were compared with the expected values. Age‐specific cutoffs were also determined using a methodology similar to previous studies.

**Result:**

Among the participants (72.5% females, median age 65 (IQR: 56.0‐69.0), median Montreal Cognitive Assessment 27 (IQR: 25.0‐28.0), median Mini‐Mental State Examination 29.0 (IQR: 28.0‐30.0) and median education level 16.0 years (IQR: 16.0‐18.0)), plasma NfL levels exceeded the 95th percentile reference from Simrén et al. in 12% (95% CI: 8.1‐16.9%) and Bornhorst et al. in 9% (95% CI: 5.7‐13.4%). These proportions, along with their confidence intervals, surpassed the expected values of 5%. Quantile regression was employed to provisionally visualize the age‐specific NfL threshold among the Thai population (Figure 1).

**Conclusion:**

Preliminary findings suggest that age‐specific cutoff values established from healthy Caucasian populations may not be universally applicable in Thailand. Factors such as ethnicity and social determinants of health may introduce confounding factors to the blood levels or pose negative effects on brain ageing. Given the increasing significance of NfL as a biomarker for various neurological diseases, there is a critical need to establish reference values for plasma NfL in diverse settings, particularly in lower‐income countries.